# Microbial Community and Function-Based Synthetic Bioinoculants: A Perspective for Sustainable Agriculture

**DOI:** 10.3389/fmicb.2021.805498

**Published:** 2022-03-11

**Authors:** Archna Suman, Venkadasamy Govindasamy, Balasubramanian Ramakrishnan, K. Aswini, J. SaiPrasad, Pushpendra Sharma, Devashish Pathak, Kannepalli Annapurna

**Affiliations:** Division of Microbiology, ICAR-Indian Agricultural Research Institute (IARI), New Delhi, India

**Keywords:** bioinoculants, PGPRs, plant microbial communities, novel biologicals, microbiome

## Abstract

Interactions among the plant microbiome and its host are dynamic, both spatially and temporally, leading to beneficial or pathogenic relationships in the rhizosphere, phyllosphere, and endosphere. These interactions range from cellular to molecular and genomic levels, exemplified by many complementing and coevolutionary relationships. The host plants acquire many metabolic and developmental traits such as alteration in their exudation pattern, acquisition of systemic tolerance, and coordination of signaling metabolites to interact with the microbial partners including bacteria, fungi, archaea, protists, and viruses. The microbiome responds by gaining or losing its traits to various molecular signals from the host plants and the environment. Such adaptive traits in the host and microbial partners make way for their coexistence, living together on, around, or inside the plants. The beneficial plant microbiome interactions have been exploited using traditional culturable approaches by isolating microbes with target functions, clearly contributing toward the host plants’ growth, fitness, and stress resilience. The new knowledge gained on the unculturable members of the plant microbiome using metagenome research has clearly indicated the predominance of particular phyla/genera with presumptive functions. Practically, the culturable approach gives beneficial microbes in hand for direct use, whereas the unculturable approach gives the perfect theoretical information about the taxonomy and metabolic potential of well-colonized major microbial groups associated with the plants. To capitalize on such beneficial, endemic, and functionally diverse microbiome, the strategic approach of concomitant use of culture-dependent and culture-independent techniques would help in designing novel “biologicals” for various crops. The designed biologicals (or bioinoculants) should ensure the community’s persistence due to their genomic and functional abilities. Here, we discuss the current paradigm on plant-microbiome-induced adaptive functions for the host and the strategies for synthesizing novel bioinoculants based on functions or phylum predominance of microbial communities using culturable and unculturable approaches. The effective crop-specific inclusive microbial community bioinoculants may lead to reduction in the cost of cultivation and improvement in soil and plant health for sustainable agriculture.

## Introduction

Cultivated soils are one of the most diverse microbial ecosystems, harboring bacteria, fungi, archaea, viruses, protists, and many others and supporting various biogeochemical cycles and plant growth. Soil microbial communities are critical to plant health and adapt rapidly to different abiotic and biotic stresses (Abdul Rahman et al., 2021). The soils and their microbial members provide humans with 98.8% of the plant foods we eat (FAO, 2018; Kopittke et al., 2019; Soto-Giron et al., 2021). The Food and Agriculture Organization (FAO) predicts that soil erosion could result in between 20 and 80% losses in agricultural yields due to human activities and climate change events. This erosion of topsoil could result in variable agricultural yields, depending on the soil type and the resource use pattern (Kopittke et al., 2019; Christy, 2021). The agrarian management of soils depends on many synthetic chemical inputs for increasing profitability and productivity. Unfortunately, intensive use of these chemical inputs has led to adverse environmental consequences from regional to global scales. To reduce chemical inputs and their associated undesirable effects in the soil and environment, microbial interventions as biological products are becoming an integral part of plant nutrient management programs and pest and disease management practices.

**FIGURE 1:**
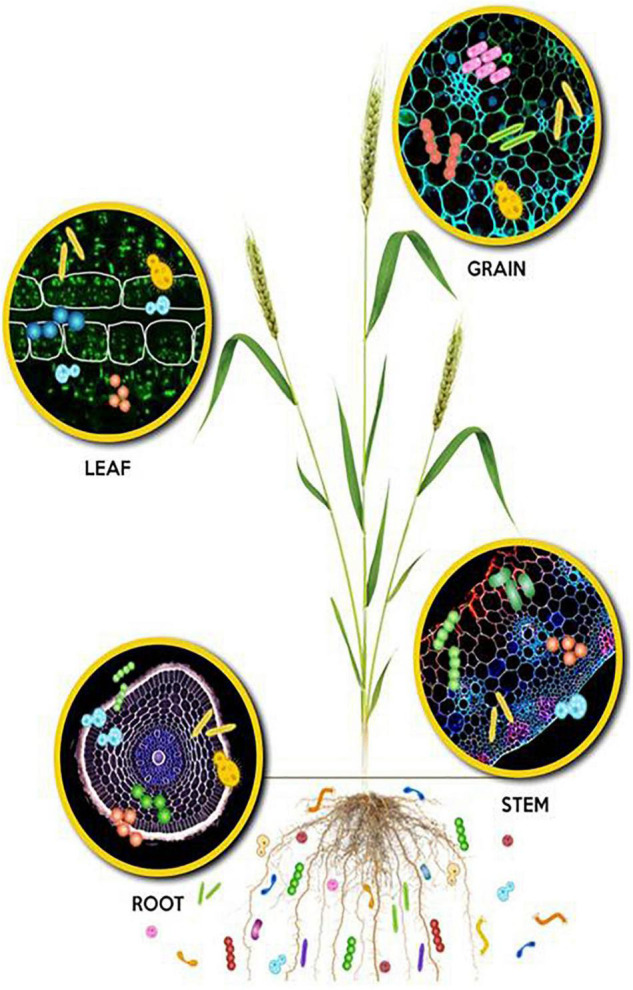
Microbial colonization depicted in different plant niches: Rhizosphere, phyllosphere and endosphere of root, stem, leaf, and grain.

Microbial communities associated with plants, presently referred to as the plant microbiome, extend the host plant genome and their functions (Figure 1). Many studies demonstrate that these microbiomes are the key determinants of plant development, health, and productivity (Conrad et al., 2006; Bulgarelli et al., 2012; Lundberg et al., 2012; Turner et al., 2013; Williams, 2013). The recent investigations have unraveled the complex network of genetic, biochemical, physical, and metabolic interactions among the plant host, the associated microbial communities, and the environment. These interactions shape the microbiome assembly and modulate beneficial traits such as nutrient acquisition and plant health (Trivedi et al., 2021). Nutrient acquisition by plants is mediated by diverse mechanisms that include (i) augmenting the surface area accessed by plant roots for uptake of water and nutrients, (ii) through nitrogen fixation, (iii) P-solubilization, (iv) the production of siderophore and HCN production, and other unknowns. Furthermore, their contributions in protection against biotic (pests and diseases) and abiotic stresses directly or through modulating intrinsic resistance/tolerance have been reported (Pii et al., 2015; Govindasamy et al., 2020; Abiraami et al., 2021). The basis of this review is to highlight strategic approaches for designing novel bioinoculants based on the plant microbiome data generated from both culturable and unculturable approaches. Such plant microbiome-based specific bioinoculants may function in a better way as compared to the conventional bioinoculants with non-specific microbial isolates. The agricultural bioinoculant market is a fast-growing sector with a compound annual growth rate (CAGR) of 6.9% with a predicted value of over 12 billion US dollars by 2025. The growth of the market is driven by increasing health concerns and awareness among consumers, resulting in the inclination toward organic farming practices or low-chemical-input agriculture. Hence, the bioinoculant technology will move forward toward reducing the cost of cultivation while improving soil and plant health for sustainable agriculture.

## Plant-Microbiome-Mediated Adaptive Functions

**FIGURE 2:**
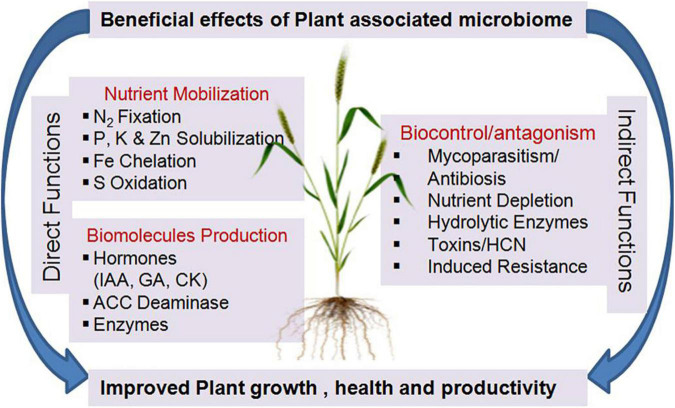
Beneficial functions of Plant associated microbiome. N, Nitrogen; P, Phosphorous; K, Potassium; Zn, Zinc; Fe, Iron; S, Sulfur; IAA, Indole Acetic Acid; GA, Giberrelic Acid; CK, Cytokinin; ACC, 1-AminoCyclopropane Carboxylate; HCN, Hydrocyanic Acid.

The microbiome is playing a significant role, throughout the plant life cycle, in altering the physiologies, and development through phytohormones, metabolites, signals, responses, nutrients, and induction of systemic resistance against pathogens as well as tolerance mechanisms against abiotic stresses such as drought, salinity, or contaminated soils (Mendes et al., 2013; Marag and Suman, 2018; Compant et al., 2019). At the community level, the microbiome functional capability is more than the sum of its individual microbial components as individual microbial species in the microbiome may interact to form a complex network, which interrelates with the host plant(s) in a mutualistic, synergistic, commensalistic, amensalistic, or parasitic mode of relationship. These interactions influence each member of the complex network for their survival, fitness, and propagation. The sum of all these interactions influences plant health *vis-a-vis* soil fertility (Berg et al., 2020). The advancement in the molecular methods and affordable sequencing has led to a greater understanding of the microbiome composition; however, translating species or gene composition into microbiome functionality still remains a challenge. Using community ecology concepts, Saleem et al. (2019) have indicated that more than individual functions, the overall microbiome biodiversity is critical as the driver of plant growth, soil health, and ecosystem functioning. By meta-analysis of numerous publications on microbial biodiversity and ecosystem functioning (BEF), they indicated that the impacts can be classified into (i) biodiversity effects (negative, no (or unknown), and positive effects of biodiversity on microbial derived services), (ii) assessed functions (nutrient cycling, protection from different stresses, etc.), and (iii) underlying mechanisms (cooperation, mutualism, etc.). Higher diversity can increase the number and resilience of plant-beneficial functions that can be co-expressed and can unlock the expression of plant-beneficial traits that are hard to obtain from any species in isolation. Therefore, the maintenance and modulation of desired microbial activities (functional pools) in the vicinity of the plant system may have more significant potential to provide crops with required nutrition and other protection systems (Figure 2).

With increasing knowledge of plant microbiome *vis-à-vis* plant performance, approaches are being devised for tapping the potential of plant-growth-promoting (PGP) isolates, by employing both culturable and unculturable approaches. The advent of “omics” technologies understandably provides the tools for a broader understanding of microbial ecosystems and their dynamic interaction with their hosts. These techniques and methods enable the screening of large microbial populations and easily identify the individual or groups of taxa with functional capabilities. Large-scale genomic analyses of plant-associated bacteria have indicated that the bacteria from phyla *Actinobacteria*, *Bacteroidetes*, *Firmicutes*, and *Proteobacteria* are dominant in different plant niches (Levy et al., 2018a,b). The exhaustive investigations on wheat seeds followed by rhizospheric, epiphytic, and endophytic bacterial diversity, growing in six diverse agro-climatic zones in India, led to more than 200 diverse bacterial isolates with PGP traits (Suman et al., 2016; Verma et al., 2016, 2019; Verma and Suman, 2018; Sai Prasad et al., 2021). The PGP rhizobacteria (PGPR) can adapt easily to adverse conditions and protect the host plants from the deleterious effects of specific environmental stresses (Glick et al., 1997). Several bacteria like *Bacillus* sp., *Azospirillum*, *Herbaspirillum*, and pink-pigmented methylotrophic bacteria have been shown to mitigate stress conditions in maize, wheat, and other crops (Chakraborty et al., 2013; Vurukonda et al., 2016; Curá et al., 2017; Ahlawat et al., 2018). Various factors related to host, microbes, and the environment influence the community composition and diversity of plant microbiome (Dastogeer et al., 2020). Our knowledge on the underlying mechanism(s) of microbiome assemblages and how they influence the host plants is still lacking. How the entire assembly of microbial communities interfere with the host fitness and health remains largely unknown. Connecting the microbiome composition comprising PGP as well as plant-growth-compromising activities and diversity to their function is a great challenge for future research.

These fundamental, microbial-mediated adaptive functions can help address the significant challenges in sustainable food production under the changing climatic conditions. Likewise, the strategic application of microbial communities rather than as individual isolates to improve plant production offers enormous potential, particularly under adverse environmental conditions. Their applications can serve multiple purposes, such as reducing climate change impact and avoiding excessive reliance on chemical fertilizers and pesticides. Earlier studies solely based on culture-dependent techniques have overlooked the benefits of collective microbial functional and genetic diversity and the advantages of the culture-independent methods (Banik and Brady, 2010; Stewart, 2012; Turner et al., 2013; De Souza et al., 2016; Waigi et al., 2017; Armanhi et al., 2018; Mourad et al., 2018).

The cultivable isolates of the microbial community members such as plant probiotics, biofertilizers, or agricultural bioinoculants have shown their distinct influences on plant growth, fitness, and stress resilience but with certain limitations. The developed formulations containing one or more beneficial microorganism strains (or species) can mediate the cycling of several elements from the soil and transform them into the more readily available form of nutrients for plant uptake. Not only do the probiotic action of these formulations increase the growth, yield, and quality of plants, but they are also a tool to produce high-quality functional foods. The use of microbial-based agricultural inputs has a long history, beginning with broad-scale rhizobial inoculation of legumes in the early twentieth century (Desbrosses and Stougaard, 2011). The “Fresh” Green Revolution, perhaps the Bio-Revolution, needs to be based on fewer intensive inputs with reduced environmental impact. It would be based on biological inputs through utilization of the phytomicrobiome (with inoculants, microbially produced compounds, etc.) and improved crops (by manipulation of the phytomicrobiome community structure) (Timmusk et al., 2017; Backer et al., 2018). With increasing data availability on plant microbiome from different ecological niches, strategic approaches based on the concomitant use of culture-dependent and culture-independent techniques, targeting all the plant-beneficial microbial groups, are necessitated to develop novel biological products in all categories like biofertilizers, biopesticides, bioagents, or bioinoculants and biostimulants.

## Potential of Bioinoculants for Field Application

The current knowledge on functions, ecological adaptations, host interactions, and putative beneficial traits of microorganisms associated with the host plants mainly revolves around a handful of cultivable rhizospheric and endophytic bacteria or fungi. Many microbial formulations having individual or mixture of strains are developed and used at present. These biological or bioinoculants are nitrogen fixers, phosphate solubilizers, siderophore producers, photohormone producers, and exopolysaccharide producers. Some of them are involved in lytic enzyme production against pests and pathogens, antibiosis, and induced systemic resistance (Gupta et al., 2015; Sruthilaxmi and Babu, 2017).

The bioinoculants are grouped as either biofertilizers or bioagents depending on the intended purpose of plant growth promotion or protection, respectively. The biofertilizers include the individual species of *Azotobacter*, *Azospirillum*, and *Rhizobium*; phosphate-, potassium-, and zinc-solubilizing bacteria; vesicular–arbuscular mycorrhiza (VAM), and *Acetobacter*. Crop-specific biofertilizers like *Gluconacetobacter diazotrophicus* for sugarcane or generic biofertilizers like *Pantoea* isolates showing multi-PGP activities in several crops have demonstrated benefits in improving crop yield and productivity (Suman et al., 2005, 2008). Not only the rhizosphere-colonizing but also several endosphere-colonizing bacteria have been exploited for their beneficial contributions in sustainable agriculture (White et al., 2019). Presently, bioinoculants are available mostly as single entities (Bashan et al., 2014) but are also being formulated as consortia with multiple bacteria and fungi, which have synergistic PGP traits for improving plant production and productivity. Tables 1, 2 summarize the current status of various microbial formulations developed using single, dual, or multiple isolates as bioinoculants to improve nutrient uptake or protect against various biotic and abiotic stresses.

**TABLE 1:** Status of various microbial inoculants developed as synthesized microbial communities in use for improving nutrient uptake and protections against plant pathogens.

S. No.	Microorganism (Bacteria)	Host/Plant associated	PGP Activity	References
	**Single culture inoculation**			

1.	*Bacillus megaterium* TRS-4	Tea	Biofertilization and biocontrol activity to reduce brown root rot disease (*Fomes lamaoensis*)	Chakraborty et al. (2006)
2.	*Pseudomonas putida* B0	Sub-alpine	Phosphate solubilisation and antagonistic activity	Pandey et al. (2006)
3.	*Pseudomonas fluorescens* GRS1	Pea	Phosphorus solubilisation and increased biomass production	Katiyar and Goel (2003)
4.	*Bacillus pumilus* ES4	Soil	Nitrogen fixation	Hernandez et al. (2009)
5.	*Azospirillum* sp. P1AR6-2	Black pepper	Phosphorus solubilisation along with improved root and shoot growth	Ramachandran et al. (2007)
6.	*Paenibacillus polymyxa* P2b-2R	Canola	Nitrogen fixation, phosphate solubilisation, antibiotic production, and other plant growth regulators for increased plant biomass	Padda et al. (2016)
7.	*Pseudomonas fluorescens* PGPR1	Peanut	Siderophore production, phosphate solubilization, increased yield and biomass production	Dey et al. (2004)
8.	*Bacillus* sp. EUCB 10	Gum trees	IAA production, phosphate solubilization, nitrogen fixation and increased biomass production	Paz et al. (2012)
9.	*Herbaspirillum seropedicae* ZAE94	Rice	Nitrogen fixation and increased biomass production	Alves et al. (2015)
10.	*Bacillus megaterium* B388	Pine	IAA production, phosphate solubilization, antagonistic activity and increased biomass production	Trivedi and Pandey (2008)
11.	*Pseudomonas fluorescens* L321	Pea	Phosphate solubilisation and increased biomass production	Otieno et al. (2015)
12.	*Bacillus aryabhattai*MDSR7	Soybean	Zinc solubilisation, decreased rhizosphere soil pH, increased dehydrogenase, glucosidase, auxin production, microbial biomass	Ramesh et al. (2014)
13.	*Acinetobacter* sp. AGM3	Rice	Zinc solubilisation and IAA production	Gandhi and Muralidharan (2016)
14.	*Bacillus megaterium* CDK25	Cow dung	Phosphate solubilization, IAA production, phytase production, siderophore production and increased plant growth	Bhatt and Maheshwari (2020)
15.	*Enterobacter cloacae* ZSB14	Rice	Zinc solubilization and increased plant growth	Krithika and Balachandar (2016)
16.	*Enterobacter* sp. MN17	Chickpea	Improved productivity, profitability, Zinc use efficiency and quality	Ullah et al. (2020)
17.	*Bacillus* sp. BPR7	Common bean	Production of plant growth regulators and antagonistic activity	Kumar et al. (2012)
18.	*Bacillus* sp. SC2b	Applegate stonecrop	ACC deaminase activity, IAA production, siderophore production, increased chlorophyll content and plant growth	Ma et al. (2015)
19.	*Burkholderia ambifaria*MCI 7	Maize	Siderophore production and antifungal activity	Ciccillo et al. (2002)
20.	*A. brasilense*Ab-V5	Maize	Nitrogen fixation and IAA production	Ferreira et al. (2013)
21.	*Rhizobium leguminosarum*bv. *viciae*	Pea	Increase in nodule number, N accumulation and nitrogen fixation	Clayton et al. (2004)
22.	*P. fluorescens*(PGPR1, PGPR2, and PGPR4)	Peanut	ACC-deaminase activity, IAA production, siderophore production, antifungal activity	Dey et al. (2004)
23.	*Azospirillum*sp. B510	Rice	Nitrogen fixation, IAA production, increase in tiller number and seed yield	Isawa et al. (2009), Bao et al. (2013)
24.	*Bacillus amyloliquefaciens*sks_bnj_1	Soybean	Siderophore production, IAA production, ACC-deaminase activity and antifungal activity, phytases production	Sharma et al. (2013)
25.	*Gluconacetobacter diazotrophicus*VI27	Sugarcane	Nitrogen fixation, siderophore production, IAA production, phosphorus solubilisation and increase in germination	Beneduzi et al. (2013)
26.	*Azospirillum brasilense*INTA Az-39	Wheat	Nitrogen fixation, IAA production and increased dry matter accumulation	Díaz-Zorita and Fernández-Canigia (2009)
27.	*A. brasilense*(Ab-V5 and Ab-V6)	Wheat and maize	Nitrogen fixation, IAA production and increased yield	Hungria et al. (2010)
28.	*Pseudomonas*sp. PS1	Mung bean	Increase plant dry weight, root nodule, total chlorophyll content, seed yield and seed protein	Ahemad and Khan (2011a,2012a)
29.	*Bradyrhizobium*sp. MRM6	Mung bean	Increased plant growth parameters	Ahemad and Khan (2011b,2012b)
30.	*Pseudomonas*sp. A3R3	Cabbage	Increased biomass production	Ma et al. (2011)
31.	*Rhizobium*sp. MRP1	Pea	Nitrogen fixation, increased nodulation, increase in N, P uptake, increase seed yield and seed protein	Ahemad and Khan (2009, 2010)
32.	*Bacillus Weihenstephanensis*SM3	Sunflower	Increased plant biomass and accumulation of trace elements like Cu, Ni and Zn	Rajkumar and Freitas (2008)
33.	Single inoculation of*Brayrhizobium diazoefficiens* USDA 110, B. Elekani USDA 61 and USDA 94	Soybean	Rhizobitoxine production, improved symbiotic effectiveness through high nodulation and nitrogen fixation under drought stress	Govindasamy et al., 2017
34.	Single inoculation of*Ochrobactrum*sp. EB-165, *Microbacterium*sp. EB-65, *Enterobacter sp.*EB-14 *and Enterobacter cloacae strain*EB-48	Sorghum	Multi-PGP traits on molecular regulation of stress responsive genes and improved physiological stress tolerance under drought	Govindasamy et al., 2020
35.	*Gluconacetobacter diazotrophicus* –IS100	Sugarcane	Efficient in promoting plant growth and N recovery more at low nitrogen input	Suman et al. (2005)
36.	*Pantoea* sp (8) *as single inoculant*	Wheat, Maize and Rice	Multi PGP generic bioinoculant for cereals	Suman et al. (2020)

	**Dual culture inoculation**			

37.	*Azospirillum brasilense* Az39	Maize	Promote seed germination, nodule formation, and early development of corn and soybean seedlings	Cassan et al. (2009)
	*Brayrhizobium japonicum* E109			
38.	*Pseudomonas fluorescens* Aur6	Rice	Most effective control against rice blast pathogen	Lucas et al. (2009)
	*Chryseobacterium balustinum* Aur9			
39.	*Bacillus subtilis* SU47	Wheat	Salinity tolerance and increased dry weight	Upadhyay et al. (2012)
	*Arthrobacter* sp. SU18			
40.	*Pseudomonas jessenii* R62	Wheat	Increased grain yield	Mäder et al. (2011)
	*Pseudomonas synxantha* R81			
41.	*Azotobacter chroococcum* A-41	Rice	Potassium solubilization, Nitrogen fixation and Mobilization of potassium-bearing minerals.	Basak and Biswas (2010)
	*Bacillus mucilaginosus*			
42.	*Bacillus subtilis* OSU-142	Chickpea	Nitrogen fixation, Phosphorus solubilisation, increased seed and total biomass yields	Elkoca et al. (2007)
	*Bacillus megaterium* M-3			
43.	*Gluconacetobacter diazotrophicus*	Sugarcane	Improves nutrient uptake (N, P and K) on inoculation with FYM	Shukla et al. (2008)
	*Trichoderma viride*			
44.	*Chryseobacterium* sp. PSR10	Soil	Phosphorus solubilization, enhanced plant growth and yield	Singh et al. (2013)
	*Escherichia coli* RGR13			
45.	*Bacillus*sp. ZM20	Bhendi	Zinc solubilisation, improved relative water content and biomass production	Fatima et al. (2018)
	*Bacillus aryabhattai* ZM31			
46.	*Pantoea dispersa* MPJ9	Mungbean	Iron chelation and increased plant growth	Patel et al. (2018)
	*Pseudomonas putida* MPJ6			
47.	*Pseudomonas aeruginosa* LSE-2	Soybean	IAA production, phosphorus and zinc solubilization, siderophore production and increased plant growth	Kumawat et al. (2019)
	*Bradyrhizobium* sp. LSBR-3			
48.	*Pseudomonas jessenii*PS06	Chickpea	Higher nodule fresh weight, nodule number and shoot N content, highest in seed yield and nodule fresh weight	Valverde et al. (2007)
	*Mesorhizobium ciceri*C-2/2			
49.	*Bacillus cereus*UW85	Soybean	Stimulations in shoot dry weight, increased seed yield and seed N content	John Bullied et al. (2002)
	*B. japonicum*			
50.	*B. japonicum* (SEMIA 5079 and SEMIA 5080)	Soybean	Nitrogen fixation, IAA production and increased yield	Hungria et al. (2013)
	*A. brasilense*(Ab-V5 and Ab-V6)			
51.	*Azospirillum*sp.	Artichoke	Increased radical, shoot length, shoot weight and increased germination	Jahanian et al. (2012)
	*Azotobacter*sp.			
52.	*Rhizobium leguminosarum*	Lentil	Improved leghemoglobin content, growth and grain yield	Singh et al. (2018)
	*Pseudomonas. fluorescens*			
53.	*Azospirillum sp. AZ204*	Cotton	Nitrogen fixation, Phosphorus solubilisation and biocontrol activity	Marimuthu et al. (2013)
	*Pseudomonas fluorescens Pf1*			
54.	*Enterobacter cloacae*	Mung bean	Increase salt tolerance, seed yield, dry biomass, plant height, leaf area, relative water content and chlorophyll	Mahmood et al. (2016)
	*Bacillus drentensis*			
55.	*Gluconacetobacter* sp.	Rice	Higher phosphatase activity, increased P uptake, increased biomass, yield, number of panicles and seeds/panicles.	Stephen et al. (2015)
	*Burkholderia* sp.			
56.	*Pantoea cypripedii*	Maize, Wheat	Increased grain yield, P uptake, shoot and root biomass	Gurdeep and Reddy (2015)
	*Pseudomonas plecoglossicida*			
57.	*Ochrobactrum ciceri*	Kabuli and Desi chickpea	Increased nodulation, biomass and grain yield	Imran et al. (2015)
	*Mesorhizobium ciceri*			

	**Triple culture inoculation**			

58.	*Gluconacetobacter azotocaptans* DS1	Maize	Alcohol production, IAA production, phosphorus solubilization, nitrogen fixation and increased biomass production	Mehnaz and Lazarovits (2006)
	*Pseudomonas putida* CQ179			
	*Azospirillum lipoferum* N7			
59.	*Bacillus thuringiensis* KR-1	Kudzu	HCN production, IAA production and increased biomass production	Selvakumar et al. (2008)
	*Enterobacter asburiae* KR-3			
	*Serratia marcescens* KR4			
60.	*Bacillus cereus* PK6-15	Guinea grass	Zinc solubilization, ammonia production, nitrogen fixation, phosphorus solubilisation and increased plant growth	Bokhari et al. (2019)
	*Bacillus subtilis* PK5-26			
	*Bacillus circulans* PK3-109			
61.	*Pseudomonas fluorescens* A506	Pear	Biological control against Fire blight pathogen	Stockwell et al. (2011)
	*Pantoea vagans* C9-1			
	*Pantoea agglomerans*			
62.	*Rhizobium*spp.	Chickpea	Nitrogen fixation, biocontrol activity and Phosphorus solubilisation	Elkoca et al. (2007)
	*B. subtilis*OSU- 142			
	*Bacillus megaterium*M-3			
63.	*Pseudomonas alcaligenes*PsA15	Maize	Nitrogen fixation and antifungal activity	Egamberdiyeva (2007)
	*Bacillus polymyxa*BcP26			
	*Mycobacterium phlei*MbP18			
64.	*P. fluorescens*ACC-5 (biotype G)	Pea	ACC-deaminase activity	Zahir et al. (2008)
	*P. fluorescens* ACC-14			
	*P. putida*Q-7 (biotype A)			
65.	*B. vietnamiensis*MG43	Sugarcane	Nitrogen fixation and increased biomass production	Govindarajan et al. (2008)
	*G. diazotrophicus*LMG7603			
	*H. seropedicae*LMG6513			
66.	*Bradyrhizobium japonicum*	Soybean and common bean	Nitrogen fixation and increased grain yield	Hungria et al. (2013)
	*Rhizobium tropici*			
	*Azospirillum brasilense*			
67.	*Rhizobium leguminosarum*	Common bean	Increased grain yield	Kumar et al. (2016)
	*Bacillus sp.*			
	*Pseudomonas sp.*			
68.	*Pseudomonas aeruginosa*	Tomato	Increased root and shoot length, ACC deaminase activity, IAA production, phosphate solubilization and siderophore production	Tank and Saraf (2010)
	*Pseudomonas uorescens*			
	*Pseudomonas stutzeri*			
69.	*Xanthomonas sp. WCS2014-23*	Arabidopsis	Less fungal spores and higher plant fresh weight	Berendsen et al. (2018)
	*Stenotrophomonas sp. WCS2014-113*			
	*Microbacterium sp. WCS2014-259*			

	**Multiple culture inoculations**			

70.	*Exiguobacteriumaurantiacum* MS-ZT10, *Trabusiella* sp. MS-ZT1, *Aeromonas*sp. MS-ZT4, *Arthrobacter*sp. MS-ZT5	Wheat	Zinc solubilisation, enhanced N, P, and K concentration	Shaikh and Saraf (2017)
71.	1:1:1:1 ratio of Proteobacteria, Actinobacteria, Bacteroidetes and Firmicutes	Arabidopsis	Reciprocal relocation between root and leaf microbiota members and functional overlap in the communities with improved plant growth	Bai et al. (2015)

	**Triple culture inoculation**			

72.	*Bacillus amylolquifaciens, Bacillus simplex*,MCP of 12 isolates *Azotobacter vinlandii*, *Clostridium* sp., *Lactobacillus* sp., *Bacillus velezensis*, *Bacillus subtilis* (SILo Sil® BS), *Bacillus thuringiensis*, *Pseudomonas fluorescens*, *Acetobacter*, *Enterococcus*, *Rhizobium japonicum*, *Nitrosomonas*, and *Nitrobacter*, as well as fungi: *Saccharomyces*, *Penicillium roqueforti*, *Monascus*, *Aspergillus oryzae*, *Trichoderma harzianum* (TRICHOSIL®), and algae extracts from *Arthrospira platensis* (Spirulina) and *Ascophyllum nodosum*	Tomato	Improved phosphate (P) acquisition, increased biomass production and fruit yield	Bradáčová et al. (2019)
73.	*Arthrobacter nitroguajacolicus E46, Bacillus mojavensis K1, Pseudomonas frederiksbergensis A176, Arthrobacter nitroguajacolicus E46, Bacillus cereus CN2, Bacillus megaterium B55, Bacillus mojavensis K1, Pseudomonas azotoformans A70, Pseudomonas frederiksbergensis A176, Pseudomonas azotoformans A70*	Tobacco	Increased fitness and survival of tobacco plants	Santhanam et al. (2015)
74.	*Bacillus megaterium SOGA_2, Curtobacterium ceanosedimentum SOGA3, SOGA6, Massilia aurea SOGA7, Pseudomonas coleopterorum SOGA5, 11, 12, Pseudomonas psychrotolerans SOGA13, Pseudomonas rhizosphaerae SOGA14 and 19, Frigoribacterium faeni SOGA17, Xanthomonas campestris OGA20*	Tomato	Fewer pathogen (*Pseudomonas syringae* pv. tomato) DNA copies in the phyllosphere of field-grown tomato plants	Berg and Koskella (2018)
75.	*8 Pseudomonas spp.*	Pea, wheat, etc.	Reduced disease severity and pathogen (*Ralstonia solanacearum*) abundance in pea, wheat, cotton, tomato, sugar beet and tobacco	Hu et al. (2016)
76.	*Pseudomonas*spp., *Bacillus amyloliquefaciens*, *Bacillus subtilis*, soil yeast	Rice	Increased grain and straw yields, total N uptake, as well as grain quality in terms of N percentage	Cong et al. (2009)
77.	*Rhizobium*, *Sinorhizobium*, *Bacillus*, *Burkholderia*	Pigeon pea	Increased plant biomass and nodule mass per plant	Pandey and Maheshwari (2007)
78.	*Arthrobacter nitroguajacolicus*, *Bacillus cereus*, *Bacillus megaterium*, *Bacillus mojavensis*, *P. azotoformans*, *P. frederiksbergensis*	Tobacco	Reduced disease incidence and mortality without influencing growth or herbivore resistance	Verma et al. (2013)
79.	Mixes of various *Pseudomonas*, *Enterobacter* and *Serratia* strains	Rapeseed	Increased rapeseed oil and grain yields	Lally et al. (2017)
80.	Various consortia involving *Enterobacter*, *Serratia*, *Pseudomonas*, *Microbacterium* and *Achromobacter*	Avocado	Mitigate water shortage and salt stress	Barra et al. (2016)
81.	*Bacillus amyloliquefaciens*strains	Tomato	Decreased disease incidence	Wei et al. (2011)
82.	*Pseudomonas spp. CHA0, PF5, Q2-87, Q8R1-96, 1M1-96, MVP1-4, F113, Phl1C2*	Pea	Reduced disease severity and pathogen abundance in pea, wheat, cotton, tomato, sugar beet and tobacco	Hu et al. (2016)
83.	4 Small communities each of endophytes from sugarcane, maize, brassica and wheat	Wheat-maize cropping system	Improves system productivity at low input of nitrogen and irrigation managing abiotic stress	Suman et al. (unpublished)

**TABLE 2:** Fungal inoculants developed as synthesized microbial communities used for improving nutrient uptake and protections against plant pathogens.

Sr No.	Microorganism (fungal)	Host/plant associated	PGP activity	References
	**Single-culture inoculation**			

1.	*Glomus* sp. 88	Wheat	Phosphorus solubilization	Singh and Kapoor, 1999
2.	*Penicillium rugulosum* IR-94MF1	Maize	Phosphorus solubilization	Reyes et al., 2002
3.	*Eupenicillium parvum* NRRL 2095	Tea	Phosphorus solubilization and high stress levels of aluminum and iron desiccation	Vyas et al., 2007
4.	*Trichoderma harzianum*	Soil	*Trichoderma*-enriched compost extracts, symbiotic association, and suppression of fungal infections	Siddiqui et al., 2008
5.	*Trichoderma asperellum* Q1	Cucumber	Siderophore production and inducement of plant systemic resistance (broad spectrum), resistance to plant pathogens, and plant growth promotion	Qi and Zhao, 2013

	**Dual-culture inoculation (mostly with bacteria)**			

6.	*Gluconacetobacter diazotrophicus* IS100	Sugarcane	Improved nutrient uptake (N, P, and K) on inoculation with FYM	Shukla et al., 2008
7.	*Trichoderma viride*			
	*Gluconacetobacter diazotrophicus* IS100	Sugarcane	Consortium brought economy in the use of fertilizer N by 45.2 kg ha^–1^ and also increased the yield by 6.1 t ha^–1^ compared to the control treatment	Yadav et al., 2009
8.	*Trichoderma viride*			
	*Bacillus*/*Pseudomonas*	Soil/rhizosphere	P solubilization and symbiotic association	Sharma et al., 2013
	*Aspergillus*/*Penicillium*			
9.	*Pseudomonas aeruginosa*	Soil and rhizosphere	Biocontrol agent against pathogen, pest, symbiotic association	Afzal et al., 2013
10.	*Trichoderma viride*			
	*Microbispora* sp.	Soil	ACC deaminase (stressbuster) and IAA production, N2 fixation, P solubilization, siderophore production, and symbiotic association	Glick, 2014; Souza et al., 2015
11.	*Streptomyces* sp.			
	*Trichoderma harzianum*	Tobacco	Effective *Ralstonia solanacearum* suppression at 68.2% disease incidence	Yuan et al., 2016
12.	*Glomus mosseae*			
	*Aspergillus* sp.	Common bean	Increased P uptake and N content, increased biomass, and increased nodule number	Elias et al., 2016
13.	*Penicillium* sp.			
	*Funneliformis mosseae*	Chili	Increased plant growth, dry weight, fruit yield, and nutrient concentration	Thilagar et al., 2016
	*Bacillus sonorensis*			
14.	*Pseudomonas*	Tomato	Sugar and vitamin production and increased sweetness	Bona et al., 2017
	AM fungi			

	**Triple-culture inoculation (mostly with bacteria)**			

15.	*Pseudomonas reactans*	Soil	N fixation and symbiotic association	Moreira et al., 2016
	*Chryseobacterium humi*			
	*Rhizophagus irregularis*			
16.	*Pseudomonas putida*	Abiotic (water) stress condition	Stimulation of plant growth, drought tolerance, IAA production, and symbiotic association	Marulanda-Aguirre et al., 2008
	*Bacillus megaterium*			
	AM fungi (*Glomus coronatum*, *Glomus constrictum*, or *Glomus claroideum*)			
17.	Two *Pseudomonas*	Tomato	Increased flowering, dimensions, and weight of tomato fruits and improved industrial and nutritional features of fruits	Bona et al., 2017
	Mixed mycorrhiza			
18.	*Pseudomonas aeruginosa* (PHU094)	Chick pea	Suppression of *Sclerotium rolfsii*	Singh et al., 2013
	*Trichoderma harzianum* (THU0816)			
	*Mesorhizobium* sp. (RL091)			
19.	*P. aeruginosa* PJHU15	Peas	Suppression of *Sclerotinia sclerotiorum*	Jain et al., 2015
	*T. harzianum* TNHU27			
	*Bacillus subtilis* BHHU100			

	**Multiple-culture inoculations (with bacteria)**			

20.	*Azospirillum*, *Rhizobium*, *Bacillus*, *Pseudomonas*, *Serratia*, *Stenotrophomonas*, *Streptomyces*, *Coniothyrium*, *Ampelomyces*, *Trichoderma*	Soil	Soil conditioner, plant pathogen suppressor, biofertilizer, plant straightener, phytostimulator, biopesticide, and symbiotic association	Berg, 2009

Although the biofertilizer/bioinoculant technology has grown into a proven biological or biotechnological innovation, it is still struggling to get acceptability and popularity with farmers, the end-users. The availability and quality of bioinoculants and their inconsistent performances under field conditions have been identified as significant issues in their adoption by the farming community (Martínez-Hidalgo et al., 2019), which requires the attention of the policymakers in different countries. Along with the development, large-scale production, and assured quality of bioinoculants, one of the most promising ways to increase their efficacy is by introducing effective delivery systems. The farmers may repose the faith, buy these products confidently, and compare their usefulness and cost–benefit ratios with conventional fertilizer inputs. Many studies on bioinoculant development and laboratory-based and field studies proving their worth indicate that these microbial resources must be considered a partial replacement as the application of chemicals may not be wholly replaceable or transferable into biologicals or microbials (Sessitsch et al., 2019).

## Designing Targeted Synthetic Bioinoculants

The natural microbial communities are composed of a mix of microbes with often unknown functions. A promising way to overcome the difficulties associated with studying natural communities is to create artificial synthetic communities that retain the key features of their natural counterparts. With reduced complexity, synthetic microbial communities behave like a defined system and can act as a model system to assess the role of key ecological, structural, and functional features of communities in a controlled way (Großkopf and Soyer, 2014).

The existing thought process of top-down and bottom-up approaches for synthesizing microbial communities is based on the functional character of the individual microbial isolate and metabolic interactions among isolates, respectively. Basic motifs of commensalism, competition, predation, cooperation, and amensalism are the key metabolic interactions for the common substrate or metabolites leading to the community formations (Großkopf and Soyer, 2014). Several reviews have summarized the study of ecological interactions among microbes in synthetic as well as in natural microbial communities (Faust and Raes, 2012; Mitri and Richard Foster, 2013). Linking the composition of microbial communities with the functions is a central challenge in microbial ecology. It may be linked in some systems, but not in others, as some functions are restricted to certain taxa (e.g., sulfate reduction), but other functions are widespread across diverse groups (e.g., photosynthesis). A microbiome may contain both phylogenetic and functional redundancy. Many novel insights on the microbial community composition and organization of plant microbiomes of several crops have come from metagenomic studies using high-throughput sequencing (Edwards et al., 2015; Beckers et al., 2016; Wagner et al., 2016). Metagenomics enables the study of all microorganisms, cultured or not, through the analysis of genomic data obtained directly from an environmental sample, providing knowledge of the species present and information regarding the functionality of microbial communities in their natural habitat. Functional metagenomics has been utilized, with much success, to identify many novel genes, proteins, and secondary metabolites such as antibiotics with industrial, biotechnological, pharmaceutical, and medical relevance (Culligan and Sleator, 2016).

A microbiome may contain both phylogenetic and functional redundancy. Phylogenetic redundancy occurs when multiple OTUs from the same lineage are present in a microbiome, while functional redundancy occurs when multiple OTUs perform the same action (e.g., nitrogen fixation) within a microbiome (Shade and Handelsman, 2012). Phylogenetic redundancy is important for defining the core microbiome, which may buffer the ecological disturbances and enable the recovery of community functions. Several reports on human microbiome indicate that gut microbiome disturbances due to heavy antibiotics are restored due to the redundancy of the core group only (Antonopoulos et al., 2009). It carries relevance in agriculture as different agri-management systems lead to the disturbances in soil microbiome *vis-a-vis* plant microbiome. Recently, Berg et al. (2021) summarized the effects of microbial inoculants on the indigenous plant microbiome and termed this unexplored mode of action as “microbiome modulation.”

Synthetic microbial community analysis in gnotobiotic systems is a valuable approach to create reproducible conditions to experimentally test microbial interactions *in situ*. Such systems have been developed for animal and plant models including the well-studied plant *Arabidopsis thaliana*. With established huge volume of data on the metagenome of different crops, there is a need for its translation to certain tailored microbiome-based solutions for promoting plant growth under a range of environmental conditions and increasing resilience to biotic and abiotic stresses. The genomic data with taxonomic status, habitat compatibility, and functional trait knowledge including metabolic potential of plant microbiome communities can be followed as the approach for designing effective microbial inoculants. Here, based on phylogenetic or functional redundancy, two approaches for synthesizing microbial-communities-based bioinoculants are discussed.

### Community-Based SB

Microbial colonization in the plant root rhizosphere is the outcome of the interplay between roots exuding chemical compounds that microbes capture as signals and on which their survival and perpetuance depend. The differential abundance of colonizing microbes and the establishment of core-microbiome-based microbial communities forms the basis for plant–microbe interactions. The core members remain present throughout the development of the crop, which may be joined by other taxa during the crop growth. The metagenome data about the relative abundance of colonizing phyla/taxa and core microbiome in the plant rhizosphere and endosphere form the basis for developing Community-Based SB (CSB). Microbial isolates representing the abundant phyla can be sourced either from the crop associated culture bank or with targeted culturomics, for developing the synthetic community. The isolates are expected to be rich in community-forming characteristics like motility, chemotaxis ability, quorum sensing, metabolic diversity, and others. This approach is a direct microbiome manipulation where inoculated CSB may serve to reduce the time required for the rhizosphere microbiome to achieve niche saturation and competitive exclusion of pathogens (Bakker et al., 2012).

Taye et al. (2020) reported that in field-grown *Brassica napus*, rhizosphere core genera found at each growth stage were generally part of the overall core taxa at the 75% prevalence threshold. *Arthrobacter*, *Bradyrhizobium*, and an unclassified *Acidobacteria* in the class Ellin6075 were present in all growth stages, while other genera joined at the flowering or harvesting stage, as the recruitment of the microbiome is governed majorly by the host plant. Metagenome analysis of more than 600 *Arabidopsis thaliana* plants from eight diverse, inbred accessions growing at different locations indicated that the core endophytic microbiome is less diverse than their corresponding rhizosphere soil microbiomes. The soil types influenced the microbial communities in the *A. thaliana* rhizosphere, but the endophytic communities were overlapping and less complex with maximum of actinobacteria and selected proteobacteria. Lundberg et al. (2012) concluded that the host plants influenced the bacterial colonization in the rhizosphere which varied between inbred lines of *Arabidopsis*, but in the endophytic compartment, it remained consistent across different soil types. An extensive bacterial culture collection that captures a large part of the natural microbial diversity of healthy *A. thaliana* plants was established (Bai et al., 2015). Carlström et al. (2019) conducted dropout and late introduction experiments by inoculating *A. thaliana* with synthetic communities from a resource of 62 native bacterial strains to test how arrival order shapes community structure and indicated that individual Proteobacteria (*Sphingomonas* and *Rhizobium*) and Actinobacteria (*Microbacterium* and *Rhodococcus*) strains have the greatest potential to affect community structure as keystone species.

Similar influences of maize inbred lines growing in different soils and agri-management systems suggested the substantial variation in α- or β-bacterial diversity and relative abundances of taxa with a small proportion of heritable variation across fields. Despite significant differences between the microbial community profiles of maize inbreeds, the estimated α- and β-diversity could not define the kinship of the 27 maize inbreeds to supplement the diversification history of maize (Peiffer et al., 2013). Edwards et al. (2015) resolved the distinct nature in the microbiomes associated with rhizosphere, rhizoplane, and endosphere of rice roots, influenced by the growing conditions and genotypes.

The functional diversity within microbial communities enables metabolic cooperation toward accomplishing more complex functions than those possibly exhibited by a single organism. The consortium members or communities can communicate by exchanging metabolites or molecular signals to coordinate their activity through temporal and spatial expression and further execution of required functions. In contrast with monocultures, microbial members at the community level can self-organize to form spatial patterns, as observed in biofilms or soil aggregates. This self-organization enables them to adapt to the gradient changes, improve resource interception, and exchange metabolites more effectively (Zhang and Wang, 2016; Ben Said and Or, 2017). Hence, the selection and sourcing of microbial members are very important for the construction of CSBs, and they can be from the microbial communities specific to plant niches like rhizosphere (Huang et al., 2018), endosphere, and phyllosphere (Kong and Glick, 2017). Kong et al. (2018) reviewed the strategies for developing synthetic microbial consortium (SMC) and suggested that the crops with good quality can be a good origin of SMC. Based on next-generation sequencing and network analysis, the core microbes can be isolated from the rhizospheric soils or the plant roots using the web-based platform KOMODO (Known Media Database). Herrera Paredes et al. (2018) designed synthetic bacterial communities based on predominant phyla and demonstrated their effect on developing specific and predictable phenotypes in *A. thaliana*. Using the plant–bacterium binary-association assays, the effect of bacterial community manipulation was observed on the plant response to phosphate (Pi) starvation. This approach might contribute to microbial communities’ rational design and deployment to improve the host response to biotic and nutritional stresses.

**FIGURE 3:**
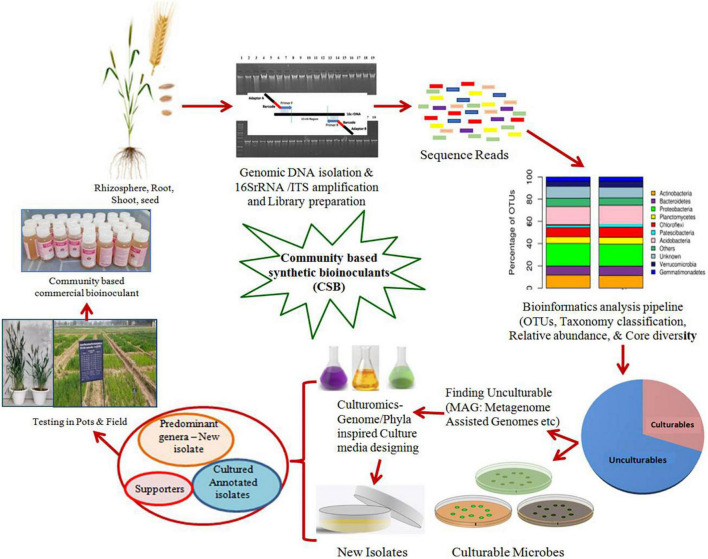
Schematic depiction of different steps for the development of microbial community based synthetic bioinoculants (CSB) by employing metagenomic and bioinformatic techniques.

*In vitro* techniques have demonstrated that the host genotypes and abiotic factors influence the composition of plant microbiomes. At the *in vivo* level, it is a challenge to define the mechanisms controlling the community dynamicity, its assembly, and the beneficial effects on the plant hosts. In an earlier study, the host-mediated natural selection of bacteria by maize roots was employed to select a simplified synthetic bacterial community consisting of seven strains (*Enterobacter cloacae*, *Stenotrophomonas maltophilia*, *Ochrobactrum pituitosum*, *Herbaspirillum frisingense*, *Pseudomonas putida*, *Curtobacterium pusillum*, and *Chryseobacterium indologenes*) representing the dominant phyla such as Proteobacteria and Actinobacteria (Niu et al., 2017). By assessing the functional role of these bacterial community combinations using axenic maize seedlings, *E. cloacae* was identified as the keystone member in this model ecosystem. This model community inhibited the phytopathogenic fungus *Fusarium verticillioides*, both *in vitro* and *in planta*, indicating a stronger benefit to the host plant. The reductionist approaches to disentangle the inherent complexity of microbial communities’ interactions have also been suggested for SynComs to be used as inoculants for a given host to decipher their key functions under the gnotobiotic system (Vorholt et al., 2017). Thus, these recent reports support the strategy of combining unculturable and culturable methods, giving the possibility of assembling a representative, yet simplified, bacterial synthetic communities from the pool of dominant genera present in the system. Figure 3 represents an outline for developing CSB based on the metagenome data and bioinformatic applications for predominant taxa and core microbiome. The key functions for developing such communities are collection of available individual isolates representing predominant taxa or isolating them using culturomic tools. Furthermore, such communities can be strengthened by their ecological interactions and probable functional annotations under gnotobiotic conditions.

### Function-Based SB

**FIGURE 4:**
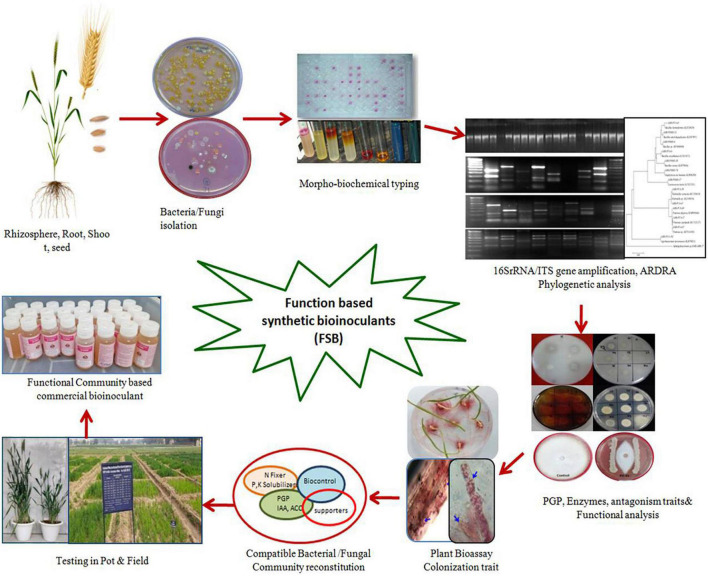
Schematic depiction of different steps for the development of microbial Function based Synthetic Bioinoculants (FSB) using functional characteristics of cultured isolates.

Due to high organic matter, soils with dynamic microbial ecologies typically have lower fertilizer requirements than conventionally managed soils (Bender et al., 2016). Focusing on the functional groups of microorganisms rather than on taxonomic relatedness and manipulating their activities (functional pools) in the vicinity of the plant ecosystem have more significant potential for providing nutrients and stress protection requirements of crops. Further exploration into the mechanisms and specificity of plant growth promotion from these key microorganisms will refine their specific use and maximize the potential inherently possessed by the microbiomes of plants or soils (Parnell et al., 2016). As only a limited proportion of microbial diversity is cultured, there is much scope for culturomics to identify, culture, and include important taxa for their beneficial exploitation (Sarhan et al., 2019). Few commercial products have emerged that take advantage of combining different biofertility products. A bacterial consortium Mammoth P™ consisting of *Comamonas testosteroni*, *P. putida*, *E. cloacae*, and *Citrobacter freundii* has been reported to enhance phosphate mobility and improve crop productivity twofold (Baas et al., 2016). The combined abilities of *Bacillus amyloliquefaciens* and the filamentous fungus *Trichoderma virens* marketed under the trade name QuickRoots^®^ (Monsanto BioAgAlliance, 2015), when applied to field corn, show positive yield improvements ranging from 220 to 500 kg ha^–1^. Similarly, several microbial consortia have been reported to improve host plants’ nutrition (Shukla et al., 2008; Suman et al., 2008; Dal Cortivo et al., 2018). The synthetic microbial community of *P. putida* KT2440, *Sphingomonas* sp. OF178, *Azospirillum brasilense* Sp7, and *Acinetobacter* sp. EMM02 has been shown to improve drought stress tolerance in maize (Molina-Romero et al., 2017). Two synthetic microbial communities (SynComs 1 and 2) of known antagonistic *Bacillus* and other isolates from compost-rich soils inhibited *Fusarium* wilt symptoms and promoted tomato growth (Tsolakidou et al., 2019). Menéndez and Paço (2020) have explored synergies between rhizobial and non-rhizobial bacteria for beneficial effects on different crops. Woo and Pepe (2018) described *Trichoderma* and *Azotobacter* as anchorage microorganisms for developing their respective consortia for promoting plant health and mitigating stress conditions. The established arbuscular mycorrhizal fungi (AMF) system, mainly known for P transport, is also a carrier of endophytes in the plant system, can induce systemic resistance to pathogens, and assists in moisture conservation (Cameron et al., 2013; Rouphael et al., 2015). Through the genomic approach of using multiplex amplicon sequencing of the community-based culture collection, Xu et al. (2016) identified the four most representative genera, *Bacillus*, *Chitinophaga*, *Rhizobium*, and *Burkholderia*, for the development of bioinoculants. Armanhi et al. (2018) gave a novel methodology for developing a PGP community-based culture collection (CBC) from sugarcane microbiomes, particularly roots and stalks. The CBC recovered 399 unique bacteria, representing 15.9% of the rhizosphere core microbiome and 61.6–65.3% of the endophytic core microbiomes of sugarcane stalks. This synthetic community of highly abundant genera was tested for colonization of maize as the test crop. The inoculated synthetic community efficiently colonized plant organs (53.9%) and improved plant biomass production, indicating their beneficial effects. Hence, the steps for designing Function-Based SB (FSB) essentially involve identifying and culturing the core microbes, selecting the microbes for plant growth functions, optimizing the microbial interactions according to their compatibility and suitable conditions, and assessing the efficacy of these FSBs under *in vitro* and *in vivo* conditions for the final release of the formulated product for farmers (Figure 4). Therefore, the FSBs can be foreseen as a small subset of the community from the natural existing microbial communities. Although the FSB may be similar to many other microbial consortia used in different crops, the fundamental difference lies in the functional analysis of the microbiome and the subsequent selection and formulation.

## Harmony of Bioinoculants With Sustainable Agriculture Goals

The UN framework of the “2030 Agenda” for 17 Sustainable Development Goals (SDGs) has been adopted by the 193 member states to develop their vision, strategy, and targets for achieving SDGs by effectively making them part of their policies. In its sustainability framework to realize the goal of ending hunger (SDG2), India has several initiatives that include the management of soil health. Successful organic cultivation and integrated agriculture will be highly dependent on the efficient microbiome-based bioinoculants for plant nutrient management and, more importantly, the recycling of crop residues for soil health (Vision 2030, DARE, India). In contrast, many other practices affect the abundance of microbial taxa involved in pest and soil disease suppression and nutrient cycling (Lupatini et al., 2017). The importance of microbiome-based solutions is gaining attention in the interrelated systems of environmental management, sustainable food, and fuel production, and human/animal health (FAO, 2019). There is a strong need for integrated research among soil and microbial scientists, growers, extension clienteles, ecologists, and policymakers to develop strategies to preserve and utilize microbial resources for soil health and crop production (Saleem et al., 2019). The microbiome research also leads to a paradigm shift in preserving axenic samples in culture collections to preserving complex communities such as “microbiome biobanks” with their functional perspectives (Ryan et al., 2021). D’Hondt et al. (2021) have summarized the key role of microbiomes in contributing policies interfacing the SDGs globally and emphasized the investments, collaborations, regulatory changes, and public outreach for innovations in microbiome-based bioeconomies.

## Conclusion

The sustainability of the modern agriculture system is critical to feed the continuously growing human and animal populations, wherein the guided use of microbiomes has an inevitable role in promoting plant growth, development, productivity, and nutrient value. The current biofertilizers are based on individual bacterial cultures with specific traits such as N fixation or the solubilization of P or K. But with the detailed diversity and functional analyses of plant-associated microorganisms, a better understanding has emerged that the plant-associated microbiomes have a tremendous and so-far untapped potential to improve the acquisition of nutrients and resilience to abiotic and biotic stresses and, ultimately, the crop yields. The options of generating synthetic communities using taxonomy abundance alone or with functionally annotated predominant taxa are now available for the improved use of microbial resources in crop cultivation. Nevertheless, developing any microbial community requires a collection of promising functionally annotated and compatible isolates in hand, rather than only microbiome data. Hence, it will be appropriate to holistically use the knowledge of unculturable microbiome generated through structural and functional genomics tools and culturable approaches to get the common and rare taxa for synthetic community preparations. The rational workflow for developing community and function-based bioinoculant preparations has been described, which can be used for developing formulations with the targeted functions of nutrient supplementation and stress management in sustainable agriculture.

## Author Contributions

AS conceptualized and wrote the manuscript. VG helped in the finalization of tables and figures. KAn and BR gave intellectual input and edited the manuscript. KAs, JS, PS, and DP contributed in data search for the content and table formulation. All authors contributed to the article and approved the submitted version.

## Conflict of Interest

The authors declare that the research was conducted in the absence of any commercial or financial relationships that could be construed as a potential conflict of interest.

## Publisher’s Note

All claims expressed in this article are solely those of the authors and do not necessarily represent those of their affiliated organizations, or those of the publisher, the editors and the reviewers. Any product that may be evaluated in this article, or claim that may be made by its manufacturer, is not guaranteed or endorsed by the publisher.
